# Cardiac metabolomic profile of the naked mole-rat—glycogen to the rescue

**DOI:** 10.1098/rsbl.2019.0710

**Published:** 2019-11-27

**Authors:** Chris G. Faulkes, Thomas R. Eykyn, Dunja Aksentijevic

**Affiliations:** 1 School of Biological and Chemical Sciences, Queen Mary University of London, G.E. Fogg Building, Mile End Road, London, UK; 2 Department of Imaging Chemistry and Biology, School of Biomedical Engineering and Imaging Sciences, King's College London, St Thomas Hospital, London, UK

**Keywords:** naked mole-rat, cardiac metabolism, metabolomics, glycogen, hypoxia

## Abstract

The African naked mole-rat (*Heterocephalus glaber*) is unique among mammals, displaying extreme longevity, resistance to cardiovascular disease and an ability to survive long periods of extreme hypoxia. The metabolic adaptations required for resistance to hypoxia are hotly debated and a recent report provides evidence that they are able to switch from glucose to fructose driven glycolysis in the brain. However, other systemic alterations in their metabolism are largely unknown. In the current study, a semi-targeted high resolution ^1^H magnetic resonance spectroscopy (MRS) metabolomics investigation was performed on cardiac tissue from the naked mole-rat (NMR) and wild-type C57/BL6 mice to better understand these adaptations. A range of metabolic differences was observed in the NMR including increased lactate, consistent with enhanced rates of glycolysis previously reported, increased glutathione, suggesting increased resistance to oxidative stress and decreased succinate/fumarate ratio suggesting reduced oxidative phosphorylation and ROS production. Surprisingly, the most significant difference was an elevation of glycogen stores and glucose-1-phosphate resulting from glycogen turnover, that were completely absent in the mouse heart and above the levels found in the mouse liver. Thus, we identified a range of metabolic adaptations in the NMR heart that are relevant to their ability to survive extreme environmental pressures and metabolic stress. Our study underscores the plasticity of energetic pathways and the need for compensatory strategies to adapt in response to the physiological and pathological stress including ageing and ischaemic heart pathologies.

## 1. Introduction

The naked mole-rat (NMR) (*Heterocephalus glaber*) is a mouse-sized eusocial African rodent that displays a range of unusual physiological characteristics from resistance to cardiovascular disease to extreme longevity [1–3]. Unlike other mammals, they do not conform to Gompertzian laws of age-related mortality as adults show no age-related change in mortality risk [4]. NMRs live in colonies that may number up to 300 individuals, in extensive underground burrows. Although parts of their burrows may often be normoxic, oxygen is likely to become scarce in crowded nest chambers where animals huddle together and sleep [4,5]. To date, the gaseous composition of an NMR nest has not been sampled in the wild. However, these nest chambers are often more than 0.5 m deep, and may be some distance from brief openings to the surface at the ephemeral mole-hills [6]. NMRs are thus adapted to be resistant to hypoxia and in laboratory experiments are able to survive anoxia for up to 18 min [2]. The mechanism underlying this may be their ability to switch from glucose to fructose driven glycolysis in the brain as the source of lactate. Other studies have revealed a number of cellular adaptations enabling tolerance to hypoxia in the brain [7–10]. The NMR can undergo rapid increases in metabolic rate to meet energy demand associated with digging through compacted soils in its xeric natural habitat with patchy food distribution [11]. They display a low baseline metabolic rate, and a recent study by Pamenter *et al*. [10] has shown that NMRs exhibit a clear decrease in metabolic rate in situations of acute hypoxia, emphasizing their ability to physiologically react to the prevailing conditions. NMRs also have low basal cardiac function accompanied by morphological traits such as cardiomyocyte hypertrophy, which is commonly associated with cardiac pathology in the Murinae and in humans [12–15]. However, they do not develop the cardiac disease and unlike pathologically remodelled hearts have enhanced contractile reserve upon increased demand [12–15]. What fuels these critical functional adaptations as well as what kind of metabolic adaptations and associated mechanisms render NMR hearts resistant to hypoxic injury and senescence remain unknown.

## 2. Methods

### (a) Animals

The non-breeding male adult NMRs used in this study were second-generation or more captive-born, descended from animals captured in Kenya in the 1980s. Colonies were maintained using artificial burrow systems as previously described [16]. The ages selected for this study allowed for physiological age matching between species such that both were at equivalent percentages of maximum lifespan and therefore not the same chronological age.

### (b) High resolution ^1^H nuclear magnetic resonance spectroscopy metabolomic profiling

Myocardial tissue was collected from adult, non-breeding NMRs (*n* = 5 males, body weight 37 ± 7 g, age ∼7 years), adult C57/BL6 mice (*n* = 5, Charles River, UK, male, 27 g, 9 weeks) and adult Wistar rats (*n* = 5, Charles River UK, male 450 g, 10 weeks) post-euthanasia. Frozen, weighed and pulverized hearts were subject to methanol/water/chloroform dual-phase extraction and high resolution ^1^H nuclear magnetic resonance spectroscopy (MRS) metabolomic profiling adapted from Chung *et al*. [17] (described in the electronic supplementary material).

## 3. Results

**Figure 1.**
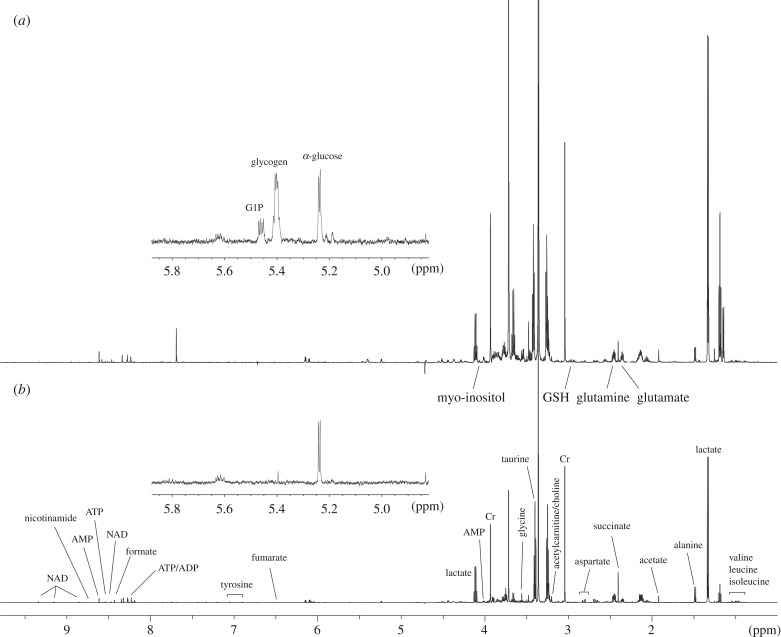
Representative cardiac ^1^H magnetic resonance spectra (MRS) of (*a*) naked mole-rat (NMR) compared to (*b*) control C57/BL6 mouse heart. Inset shows a detail in the region of the glucose and glycogen peaks.

**Figure 2.**
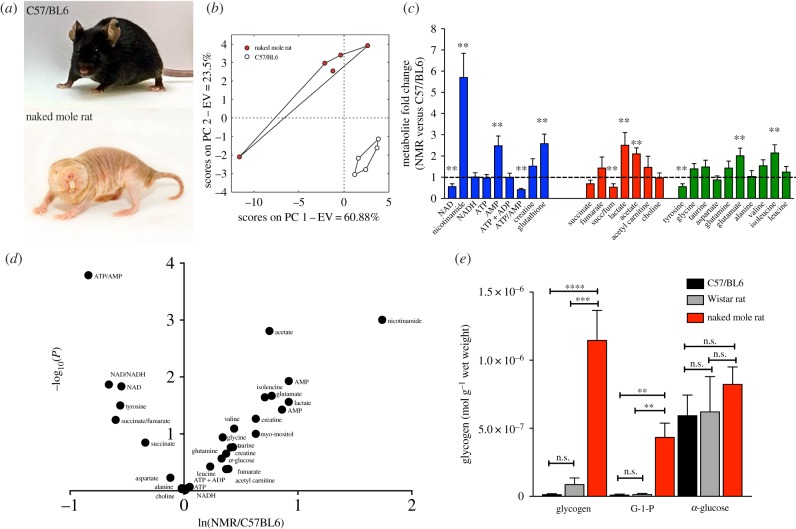
Cardiac metabolomic profile of naked mole-rats. (*a*) C57/BL6 mouse and naked mole-rat. (*b*) Principal component analysis (PCA) of NMR versus C57/BL6 cardiac metabolomic profile showing a good separation between the two groups. (*c*) ^1^H nuclear magnetic resonance spectroscopy metabolomic profile presented as fold change NMR versus C57/BL6: redox and energetics (blue bars), TCA cycle, glycolysis and lipid metabolism intermediates (red bars) and amino acid metabolism (green bars). (*d*) Volcano plot showing ln(NMR/C57/BL6) versus −log_10_(*P*). (*e*) Myocardial concentration of glycogen, glucose-1-phosphate (G-1-P) and α-glucose in C57/BL6, Wistar rat and NMR. *n* = 5/group. *p < 0.05, **p < 0.01, ***p < 0.001 versus C57/BL6.

High resolution ^1^H MRS based metabolomic analysis of NMR heart tissue (representative spectra shown in figure 1) revealed distinctly different metabolomic profiles compared to C57/BL6 hearts (figure 2*b*) and changes in several key metabolites were observed (figure 2*c*,*d*). Despite reduced contractile performance and baseline hypertrophy [12–15], NMR hearts were not energetically compromised as the levels of creatine and ATP were comparable to C57/BL6 wild-type hearts. Myocardial glycogen and glucose-1-phosphate levels (G-1-P) resulting from glycogen turnover were significantly higher in the NMR hearts and were undetectable in C57/BL6 mouse hearts (*p* < 0.001; figure 2*e*). Liver glycogen content was also found to be markedly higher in NMRs than C57/BL6 (4.1 ± 1.1 versus 0.31 ± 0.08 µmol g^−1^; *p* < 0.001). Surprisingly myocardial glycogen in the NMR was even greater than the mouse liver which is the principal glycogen storage organ with highest intracellular deposits (1.2 ± 0.02 versus 0.4 ± 0.08 µmol g^−1^; *p* < 0.001). A significant difference in myocardial glycogen levels was also observed in NMRs compared to Wistar rats, the latter was not significantly different to that found in C57/BL6 mice (thus representing another hypoxia-intolerant rodent). Glycogen regulation involves a complex interplay between multiple signalling pathways including 5′ adenosine monophosphate-activated protein kinase (AMPK) [18]. Acute AMPK activation stimulates glucose transport and glycolysis while inhibiting glycogen synthase activity. However, chronic AMPK activation has been reported to cause increased glycogen accumulation consistent with AMP levels being elevated 2.5-fold in NMR hearts (figure 2*c*; *p* < 0.01) compared to C57/BL6 mouse hearts [19].

Previous work has identified myocardial fumarate and succinate as key mediators of intracellular ROS damage during reperfusion [20,21]. In NMRs, differences in fumarate and succinate were not significant, however, an overall decrease in succinate/fumarate ratio was significant, when compared to C57/BL6. This is indicative of reduced reliance on OXPHOS and oxygen for ATP provision, and in agreement with the elevated NMR cardiac levels of intracellular lactate (2.5-fold; *p* < 0.02), indicative of higher glycolytic versus oxidative flux. There was also as twofold increase in levels of acetate (*p* < 0.01) indicative of higher pyruvate turnover [22]. Despite the regular hypoxic conditions experienced by NMRs in their colony, lack of myocardial succinate elevation could be an important cardioprotective mechanism, as succinate has been identified as a metabolic mediator of hypoxia-related ROS formation and elimination at the cross roads of several metabolic pathways [23].

Furthermore, increased glutathione levels were also observed (figure 2*c*; 2.6-fold, *p* < 0.01), which is consistent with the observations of Munro *et al.* [24,25], where ROS scavenging and antioxidant defences were found to be elevated in the NMR, conferring a protective role against oxidative stress and ROS damage [26].

Differences in amino acid metabolism were also observed. The levels of glutamate were significantly higher in NMRs (approx. twofold; *p*-value < 0.01), reflecting an alteration in anaplerotic pathways feeding the TCA cycle, while glutamate is also critical in glutathione synthesis. Reduced myocardial tyrosine levels corresponded to reduced circulating tyrosine levels previously reported in NMRs [27]. Levels of isoleucine were significantly elevated (*p* < 0.01), providing an important nutrient source. Isoleucine is also a potent signalling molecule. Branched-chain amino acid (BCAA) l-leucine is a highly effective activator of mTOR signalling, [28] a key pathway for regulation of protein synthesis in hypertrophy which is exhibited in NMR heart [29–31]. mTOR activation also triggers metabolic changes in muscle, liver and other tissues by altering insulin sensitivity[32]. Therefore, it is plausible that the elevation of local BCAA concentration observed can lead to chronic induction of cardiac mTOR activity, in turn, promoting cardiac hypertrophy observed under normal physiological conditions in NMRs.

## 4. Discussion

We applied a semi-targeted metabolomic analysis approach and identified unique metabolic signatures characterized by oxygen sparing, ROS damage reduction and readily available ATP store strategy in NMR cardiac tissue that are a likely source of cardioprotection during hypoxia and senescence. Mammalian myocardial glycogen is considered an atavistic remnant of our amphibian ancestry [33]. Myocardial glycogen is an important source of ATP under conditions of metabolic stress which would account for the ability of NMR hearts to increase their cardiac reserve upon sudden demand, and maintain low basal metabolic rates even during prolonged hypoxia or decreased nutrition. A large glycogen store is commonly observed in hypoxia-tolerant species. The role of glycogen in hypoxia resistance is most pronounced in amphibian hearts [33], which are particularly rich in this compound [34,35]. Glycogen accounts for 2% of the cell volume in the healthy mammalian adult and 30% in the fetal myocardium [33]. Even though the fetal heart has better hypoxia adaptation than the adult heart, the physiological role of glycogen remains controversial [33]. Some consider it to be the damaging lactate and proton source in the ischaemic or stressed heart, causing severe contractile dysfunction particularly upon reperfusion [33,36]. However, previous work showed that blood and tissue pH levels in NMRs are largely unchanged after acute hypoxia, suggesting the absence of a metabolic acidosis [10]. As glycogen stores are markedly increased in the myocardium of hibernating mammals [37,38] and linked to increased hypoxia survival [39], there are others attributing a cardioprotective effect to glycogen in the ischaemic heart [40–42]. Glycogen-rich hearts have been characterized by enhanced ischaemia tolerance, decreased protein loss and cell damage upon reperfusion, [33,43] leading to a hypothesis that glycogen acts as an anchoring molecule for other macromolecular cell constituents including adenine nucleotides and proteins [33,44,45] thereby integrating metabolic pathways required for myocardial survival during hypoxia [33].

Our study reveals similarities in glycogen metabolism between the hearts of NMRs and diving seals which have the ultimate physiological adaptation to prolonged and varying degrees of hypoxia [46–51] as well as Himalayan Sherpas [52]. The first lines of myocardial hypoxia defence include suppression of ATP supply and demand pathways [33], thus reducing ROS generation, during which hypoxia-tolerant systems activate protective mechanisms including metabolic reliance on intracellular glycogen [33,47,53]. When exposed to hypoxia, augmented glycogen stores would enable the NMR heart to switch to carbohydrates as the main fuel for respiration thus improving the metabolic efficiency (ATP produced per mole of oxygen consumed) [33,54], responding to acute increases in workload and stress by catabolizing their large intracellular glycogen store. This energetic advantage of glucose oxidation in the heart is well documented [33,55]. MRS and positron emission tomography (^18^FDG PET) studies in Sherpa hearts have suggested that enhanced glucose uptake, deposition and utilization in hypoxia are also advantageous for aerobic metabolism when O_2_ is present but at a premium [52]. Furthermore, the burst in glycogen oxidation is usually quickly followed by enhanced glycogen re-synthesis [54,56], and when an alternative metabolic substrate such as lactate is available for oxidation, glycogen stores are completely preserved [33,57]. Although both fatty acids and glucose are believed to be cycled through tissue-storage pools in the myocardium, the effect of such cycling has different consequences in terms of ATP provision. The incorporation of glucose into glycogen and the incorporation of fatty acids into triglycerides both consume ATP. However, in the breakdown of glycogen the energy expended in the synthesis is almost fully recovered with the efficiency of temporary storage processes close to 100% [58].

The cardioprotective effects of glycogen in NMRs could extend beyond its role as an endogenous metabolic substrate. It is plausible that cellular proteins are protected from glycosylation and in turn glucotoxicity by intracellular free glucose shunting into glycogen, comparable to shunting of fatty acid metabolites into triglycerides to protect the heart from lipotoxicity [33,59]. Furthermore, the rapid glycogen replenishment was shown to restore Ca^2+^ sensitivity and maximum Ca^2+^-activated force in glycogen-depleted skeletal muscle [60]. Glycogen and glycogen-metabolizing enzymes co-locate with sarcoplasmic reticulum [61], thus playing a major role in the complex metabolic signalling systems of calcium homeostasis and cell survival [33,46].

## 5. Study limitations

In the current study, we have investigated myocardial metabolomic differences in C57/BL6 mice and NMRs. Significant errors are known to be associated when inferring environmental adaptation from a given observation in two species where there are potential phenotypic differences between the two species at baseline, for example, due to evolution [62]. Our study was performed under basal O_2_ conditions and therefore not representative of the environmental adaptations to hypoxia *per se*. Our study, therefore, likely reflects phenotypic alterations in the two species under normoxic conditions, some of which are likely relevant to their ability to survive extreme environments such as hypoxia. However, further work will be required to elucidate the complex interplay between environmental hypoxia and evolution in these species.

A further limitation of the current study is the use of MRS for quantification of glycogen. ^1^H magnetic resonance gives a measure of the concentration of glucose monomers that are present in the observed peak normalized to the reference trimethylsilyl propanoic acid (TSP) peak. Despite being a large macromolecule, with possible differences in the mobility of glycosyl units, glycogen has been reported to be fully visible by MRS [63]. However, we used a modified dual-phase Folch extraction method used for separating aqueous and lipid metabolites which has not been optimized for the extraction of glycogen *per se*. Thus, a direct comparison of the glycogen tissue values obtained in this study to the values published by traditional enzymatic techniques may be at variance. Notwithstanding, the application of the same methods to all three species allows an assessment of fold changes between groups and therefore our assessment of between species variation remains valid.

## Supplementary Material

Supplementary Methods

## Supplementary Material

Metabolomics Data Deposition

## Data accessibility

Data are provided as an electronic supplementary material Excel file.
