# Supplementary material for: Cardiac metabolomic profile of the naked mole-rat—glycogen to the rescue
Source: Biol Lett. 2019 Nov 27;15(11):20190710. doi: 10.1098/rsbl.2019.0710 (PMC6892520; doi:10.1098/rsbl.2019.0710)
Supplement: Supplementary Methods [file rsbl20190710supp1.docx]

Faulkes, C. Eykyn, TR. Aksentijevic, D.

**Cardiac metabolomic profile of the naked mole-rat – glycogen to the rescue**

**Online Supplement**

**Materials and Methods**

*High resolution ^1^H nuclear magnetic resonance spectroscopy metabolomic profiling*

Myocardial tissue from naked mole-rats, C57/BL6 mice and Wistar rats was snap-frozen in liquid N_2_ post-euthanasia. Frozen, weighed and pulverized hearts were subject to methanol/water/ chloroform dual-phase extraction adapted from Chung et al. (1) The upper aqueous phase was separated from the chloroform and protein fractions. 20-30 mg chelex-100 was added to chelate paramagnetic ions, vortexed and centrifuged at 3600 RPM for 1 minute at 4°C. The supernatant was added to a fresh Falcon tube containing 10 µL universal pH indicator solution followed by vortexing and lyophilisation. Dual-phase-extracted metabolites were reconstituted in 600 µL deuterium oxide (containing 8 g/L NaCl, 0.2 g/L KCl, 1.15 g/L Na2HPO4, 0.2 g/L KH2PO4 and 0.0075% w/v trimethylsilyl propanoic acid (TSP)) and adjusted to pH ≈ 6.5 using 1 M hydrochloric acid and/or 1M sodium hydroxide (<5 µL of each).

Samples were analysed using a vertical-bore, ultra-shielded Bruker 14.1. tesla (600 MHz) spectrometer with a bbo probe at 303K. Nuclear magnetic resonance spectra were acquired with the Bruker noesygppr1d pulse sequence with 128 scans, 4 dummy scans and 20 ppm sweep width, acquisition time of 2.6s, pre-scan delay of 4s, 90° flip angle and experiment duration of 14.4 minutes. TopSpin (version 4.0.5) software was used for data acquisition and for metabolite quantification. FIDs were multiplied by a line broadening factor of 0.3 Hz and Fourier-transformed, phase and automatic baseline-correction were applied. Chemical shifts were normalised by setting the TSP signal to 0 ppm. Peaks of interest were initially integrated automatically using a pre-written integration region text file and then manually adjusted where required. Assignment of metabolites to their respective peaks was carried out based on previously obtained in-house data, confirmed by chemical shift and confirmed using Chenomx NMR Profiler Version 8.1 (Chenomx, Canada). Peak areas were normalized to the TSP peaks and metabolite concentrations quantified per gram tissue wet weight (1, 2). The fold change with respect to the control group was calculated for each metabolite. The propagated standard error (SEM) of the ratio was calculated using the formula ${SEM}_{(NMR/WT)}=(NMR/WT)\sqrt{{({SEM}_{NMR}/NMR)}^{2}+{({SEM}_{WT}/WT)}^{2}}$, assuming the covariance between the two groups is zero, i.e. NMR and WT are uncorrelated. Between group comparison was by Student’s t-test. Volcano plots were calculated as ln(NMR/C57BL6) vs –log_10_(P). Principle component analysis (PCA) was performed in Matlab. (3)

**References**

1. Chung YL, Leach MO, Eykyn TR. Magnetic Resonance Spectroscopy to Study Glycolytic Metabolism During Autophagy. Methods Enzymol. 2017;588:133-53.

2. Mansor LS, Mehta K, Aksentijevic D, Carr CA, Lund T, Cole MA, et al. Increased oxidative metabolism following hypoxia in the type 2 diabetic heart, despite normal hypoxia signalling and metabolic adaptation. The Journal of physiology. 2016;594(2):307-20.

3. Ballabio D. A MATLAB toolbox for Principal Component Analysis and unsupervised exploration of data structure. Chemom Intell Lab Syst. 2015;149:1-9.
